# Cyclometalated iridium(III) complexes combined with fluconazole: antifungal activity against resistant *C. albicans*

**DOI:** 10.3389/fcimb.2023.1200747

**Published:** 2023-07-21

**Authors:** Jun-Jian Lu, Zhi-Chang Xu, Hou Zhu, Lin-Yuan Zhu, Xiu-Rong Ma, Rui-Rui Wang, Rong-Tao Li, Rui-Rong Ye

**Affiliations:** ^1^ Faculty of Life Science and Technology, Kunming University of Science and Technology, Kunming, China; ^2^ College of Chinese Materia Medica, Yunnan University of Chinese Medicine, Kunming, China

**Keywords:** iridium(III) complexes, fluconazole, *C. albicans*, ROS, antifungal activity

## Abstract

*Candida albicans* (*C. albicans*) is a ubiquitous clinical fungal pathogen. In recent years, combination therapy, a potential treatment method to overcome *C. albicans* resistance, has gained traction. In this study, we synthesized a series of cyclometalated iridium(III) complexes with the formula [Ir(C-N)_2_(tpphz)](PF_6_) (C-N = 2-phenylpyridine (ppy, in **Ir1**), 2-(2-thienyl)pyridine (thpy, in **Ir2**), 2-(2,4-difluorophenyl) pyridine (dfppy, in **Ir3**), tpphz = tetrapyrido[3,2-a:2',3'-c:3'',2''-h:2''',3'''-j]phenazine) and polypyridyl ruthenium(II) complexes with the formula [Ru(N-N)_2_(tpphz)](PF_6_)_2_ (N-N = 2,2'-bipyridine (bpy, in **Ru1**), 1,10-phenanthroline (phen, in **Ru2**), 4,7-diphenyl-1,10-phenanthroline (DIP, in **Ru3**)), and investigated their antifungal activities against drug-resistant *C. albicans* and their combination with fluconazole (FLC). Of which, the combination of the lead iridium(III) complex **Ir2** and FLC showed strong antifungal activity against drug-resistant *C. albicans.* Mechanism studies have shown that they can inhibit the formation of hyphae and biofilm, damage mitochondrial function and accumulate intracellular ROS. Therefore, iridium(III) complexes combined with FLC can be used as a promising treatment to exert anti-drug-resistant *C. albicans* activity, in order to improve the treatment efficiency of fungal infection.

## 1 Introduction

*Candida albicans* (*C. albicans*) is an opportunistic pathogen (Witchley et al., 2019), which widely exists in many parts of human body. Generally, it does not cause disease, but when the body's immune function is impaired and its immune response ability is insufficient, infection will occur (Dadar et al., 2018; Celiksoy et al., 2022; Lubkin and Lionakis, 2022; Maurya and Mishra, 2022). The pathogenicity of *C. albicans* is related to both the body's immune function and its own virulence. *C. albicans* has two forms, yeast form and hyphal form (Lo et al., 1997). Yeast form hardly causes disease, when it is transformed into hyphal form, its adhesion to host epidermal cells increases sharply (Huang, 2012), and easier to cause invasive infection. Another virulence factor is the ability to form biofilms (Ponde et al., 2021), which refers to the organized fungal colony attached to the surface of living or inanimate objects and wrapped by fungal extracellular macromolecules. Biofilms are highly resistant to antibiotics and host immune defense (Crump and Collignon, 2000; Nobile and Johnson, 2015; Pohl, 2022).

*C. albicans* increases its virulence mainly by forming hyphae and biofilm. At present, the conventional drugs mainly used for the treatment of *C. albicans* infection are azole compounds (Yu et al., 2022), such as fluconazole (FLC), nystatin, etc. However, because these drugs can only play a role in inhibiting fungi, they cannot directly kill *C. albicans*. In recent years, the repeated use of antifungal drugs such as azole drugs and long-term abuse of antibiotics have led to the sharp increase of drug-resistant *C. albicans* (Prasad et al., 2019), which has dramatically decreased the therapeutic effect of *C. albicans* infection (Cowen and Steinbach, 2008; Perfect, 2017; Popp et al., 2019). The need for new drug development and new therapeutic regimens for the treatment of *C. albicans* infection has become urgent. However, due to high investment, long cycle and slow progress in the research and development of new drugs, the combination medication can well exert the synergistic effects of drugs (Li et al., 2020; Lee et al., 2021; Li et al., 2021; An et al., 2022), improve the curative effect and effectively reduce the generation of drug resistance.

In the past decade, cyclometalated iridium(III) complexes and polypyridyl ruthenium(II) complexes exhibited noteworthy applications as anticancer agents (Das et al., 2021; Pragti et al., 2021). A large number of cyclomethylated iridium(III) complexes and polypyridyl ruthenium(II) complexes have been reported as enzyme inhibitors (Păunescu et al., 2017; He et al., 2021; Zhao et al., 2023), mitochondrial targeting agents (Guan et al., 2020; Peng et al., 2020; Qin et al., 2020; Zhang et al., 2021; Yuan et al., 2023), apoptosis (Ma et al., 2019; Zhang et al., 2022), autophagy (He et al., 2014; Chen et al., 2021), or ferroptosis inducers (Wang et al., 2020; Yuan et al., 2021). Our research group has also done some work on the anti-tumor effects of cyclometalated iridium(III) complexes and polypyridyl ruthenium(II) complexes (Lu et al., 2022b; Ma et al., 2022a; Ma et al., 2023b). Recently, we have reported a series of iridium(III) and ruthenium(II) complexes with Jumonji domain-containing protein (JMJD) histone demethylase inhibitory activity (Lu et al., 2022a; Ma et al., 2022b). In addition to the research on tumors, metal complexes have also attracted widespread attention as promising candidates for addressing antimicrobial resistance and drug-resistant bacterial infections (Li et al., 2015; Frei et al., 2020; Wang et al., 2022; Li et al., 2023). However, to the best of our knowledge, most of them mainly focus on antibacterial activities, and there are relatively few reports of metal complexes with antifungal activities (Chen et al., 2018; Fu et al., 2022).

Metal complexes using tetrapyrido[3,2-a:2',3'-c:3'',2''-h:2''',3'''-j]phenazine (tpphz) as bridging ligands are focused on targeting DNA, interacting with DNA and acting as DNA imaging probes (Archer et al., 2019). These complexes are also potential phototherapeutic agents that can exert photocytotoxic effects by damaging duplex sequences (Archer et al., 2019). Being inspired by the above, and combined with the current research and development trends of antifungal drugs, we synthesized a series of tpphz modified cyclometalated iridium(III) complexes [Ir(C-N)_2_(tpphz)](PF_6_) (C-N = 2-phenylpyridine (ppy, in **Ir1**), 2-(2-thienyl)pyridine (thpy, in **Ir2**), 2-(2,4-difluorophenyl) pyridine (dfppy, in **Ir3**) and polypyridyl ruthenium(II) complexes [Ru(N-N)_2_(tpphz)](PF_6_)_2_ (N-N = 2,2'-bipyridine (bpy, in **Ru1**), 1,10-phenanthroline (phen, in **Ru2**), 4,7-diphenyl-1,10-phenanthroline (DIP, in **Ru3**)). The minimum inhibitory concentration (MIC) and fractional inhibitory concentration index (FICI) of **Ir1**-**Ir3** and **Ru1**-**Ru3** against *C. albicans* were tested. The time killing effect of the lead compound **Ir2** combined with FLC on drug-resistant *C. albicans*, their ability to inhibit biofilm formation, and the impact on morphological transformation and mitochondrial function were evaluated. Through this study, it is expected to obtain a new therapeutic strategy to improve the current therapeutic effectiveness of antifungal infections.

## 2 Results and discussion

### 2.1 Synthesis and characterization

The synthetic methods of cyclometalated iridium(III) complexes (**Ir1** (Chen et al., 2011) and **Ir3** (Cho et al., 2016)) and polypyridyl ruthenium(II) complexes (**Ru1** (Gill et al., 2011), **Ru2** (Gill et al., 2011) and **Ru3** (Alatrash et al., 2017)) were modified according the literatures, and the synthetic route was shown in **Scheme 2**. Briefly, 1,10-phenanthroline-5,6-dione was first coordinated with the precursors of cyclometalated iridium(III) or polypyridyl ruthenium(II) to obtain the intermediate products, which were then condensed with 5,6-diamino-1,10-phenanthroline to obtain the target products. Among these, **Ir2** is a newly synthesized compound. The synthesized metal complexes were characterized by ESI-HRMS, ^1^H NMR (**Figures S1**-**S12**) and elemental analysis.

### 2.2 Determination of MIC and FICI of *C. albicans* by Ir1-Ir3 and Ru1-Ru3

**Figure 1:**
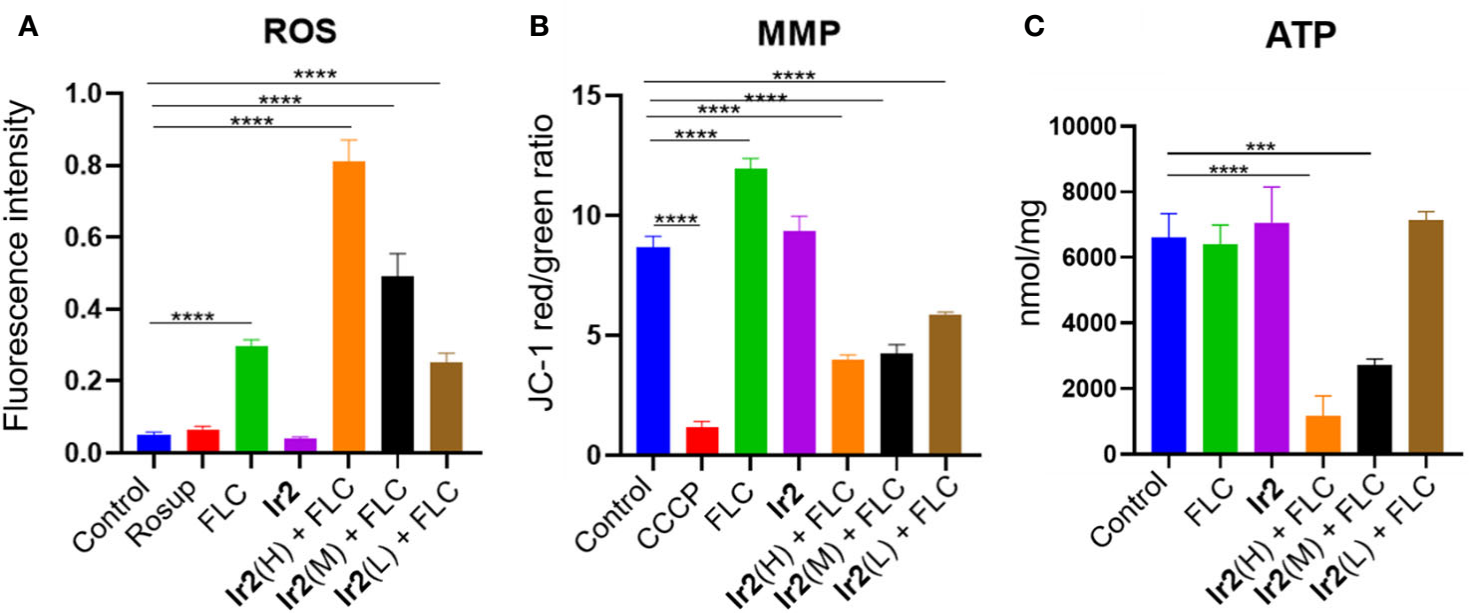
Changes of ROS **(A)**, MMP **(B)** and ATP **(C)** levels in drug-resistant *C. albicans* (****P < 0.0001 *vs* Control group, ***P < 0.001 *vs* Control group). *C. albicans* SC5314FR was incubated with FLC, **Ir2** or **Ir2** + FLC at 37 °C for 16 h. After that, the levels of ROS, MMP and ATP were measured using a multifunctional enzyme-labeled instrument.

Here, we determined the antifungal effects of **Ir1**-**Ir3** and **Ru1**-**Ru3** on *C. albicans* strains, including a sensitive strain SC5314 and a FLC-resistant strain CA23. As shown in **Table 1**, these compounds alone did not inhibit both sensitive and resistant strain of *C. albicans* (MIC > 100 μg/mL). The combination of **Ru1**-**Ru3** with FLC exhibited moderate antifungal activity against *C. albicans* susceptible strain, with MIC_50_ values of 25.32 µg/mL, 11.02 µg/mL and 8.93 µg/mL, respectively. The combination of **Ir1**-**Ir3** with FLC did not inhibit *C. albicans* susceptible strain. It has been reported that FLC exerts its antifungal mechanism by binding with ergosterol synthase on fungal cell membrane (Ahmad et al., 2010). The new newly synthesized complexes **Ir1**-**Ir3** and **Ru1**-**Ru3** did not show inhibitory effect on sensitive strains, possibly due to their inability to bind with ergosterol synthase on fungal cell membrane. The combination of **Ir1**-**Ir3** and to some extent **Ru1**-**Ru3** with FLC makes the sensitive strain not susceptible to FLC anymore, which may be due to the addition of **Ir1**-**Ir3** or **Ru1**-**Ru3** leads to the inability or weak binding of FLC to ergosterol synthase on the cell membrane of sensitive strain. While, the combination of **Ir1**-**Ir3** with FLC showed synergistic antifungal activity against resistant strain (FICI < 0.5). Notably, the combination of **Ir2** and FLC showed significant antifungal activity (MIC 2.09 μg/mL) and synergistic effect (FICI 0.02), which was the best among these combinations. Our previous research found that the change in energy metabolism of *C. albicans* was crucial to the production of FLC resistance (Li et al., 2020). Therefore, we speculate that the combination of the newly synthesized complex with FLC may affect the energy metabolism of drug resistant strains and reverse the drug resistance of FLC. This speculation has been confirmed by subsequent experiments (**Figure 1C**).

**Table 1:** The antifungal activity of **Ir1**-**Ir3** and **Ru1**-**Ru3** against the sensitive strain SC5314 and the FLC-resistant strain CA23.

Entry	Compounds	MIC_50_ (μg/mL)		FICI	
		SC5314	CA23	SC5314	CA23
1	FLC	3.41	> 100	–	–
2	**Ir1**	> 100	> 100	–	–
3	**Ir1** + FLC	> 100	10.14	–	0.10
4	**Ir2**	> 100	> 100	–	–
5	**Ir2** + FLC	> 100	2.09	–	0.02
6	**Ir3**	> 100	> 100	–	–
7	**Ir3** + FLC	> 100	10.96	–	0.11
8	**Ru1**	> 100	> 100	–	–
9	**Ru1** + FLC	25.32	57.65	7.55	0.58
10	**Ru2**	> 100	> 100	–	–
11	**Ru2** + FLC	11.02	> 100	3.29	–
12	**Ru3**	> 100	> 100	–	–
13	**Ru3** + FLC	8.93	> 100	2.66	–

Resistance of *C. albicans* is currently the main reason for the reduced efficacy of antifungal agents. We further tested the antifungal activity of **Ir2** against different resistant strains of *C. albicans*. As shown in **Table 2**, **Ir2** alone had no inhibitory effect on resistant strains of *C. albicans* (MIC > 200 μg/mL). While **Ir2** combined with FLC exhibited significant antifungal activity, with MICs of 1.88-13.27 μg/mL, and FICIs of 0.009-0.07, indicating a strong synergistic effect. Therefore, we choose compound **Ir2** as a more promising drug and the drug-resistant strain SC5314FR for further research.

**Table 2:** Antifungal activity of compound **Ir2** against different resistant strains of *C. albicans*.

Entry	Organism	MIC_50_ (μg/mL)			FICI
		FLC	Ir2	Ir2 + FLC	
1	CA23	> 200	> 200	2.09	0.02
2	CA556	> 200	> 200	2.05	0.01
3	SC5314FR	> 200	> 200	1.88	0.009
4	ATCC14053FR	> 200	> 200	8.64	0.04
5	ATCC10231FR	> 200	> 200	13.27	0.07

### 2.3 Time-kill curve of Ir2 combined with FLC

**Figure 2:**
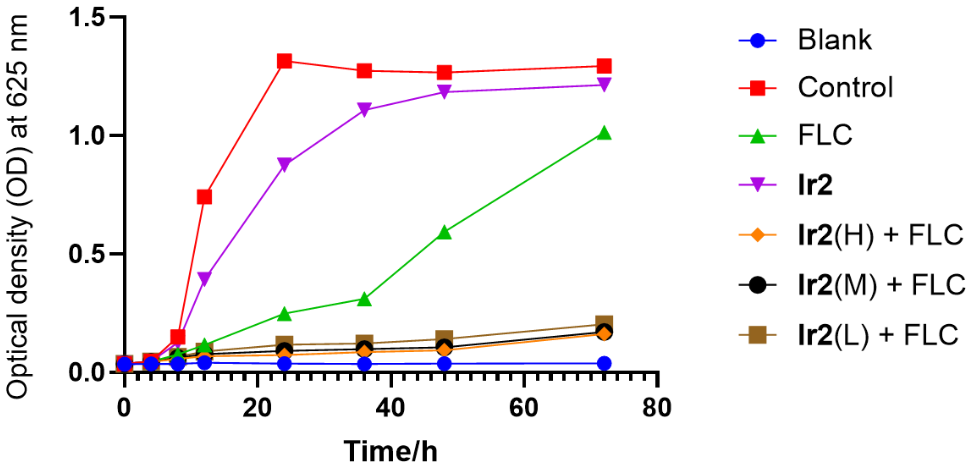
Time-kill curve of **Ir2** combined with FLC against *C. albicans* SC5314FR. **Ir2**(H) represents the **Ir2** dose of 6 μg/mL, **Ir2**(M) represents the **Ir2** dose of 3 μg/mL, **Ir2**(L) represents the **Ir2** dose of 1.5 μg/mL. The OD value at 625 nm wavelength was detected by enzyme-labeled instrument.

To evaluate the fungicidal effect of the combination of **Ir2** and FLC on drug-resistant *C. albicans* SC5314FR, a three-day concentration-dependent time-kill curve was performed. The results showed (**Figure 2**) that **Ir2** had no fungicidal effect on drug-resistant *C. albicans* SC5314FR alone, and FLC only showed a fungicidal effect within 24-36 h, which weakened with the passage of time. Only when the two drugs were used in combination, they showed a strong fungicidal effect, all combined groups still showed a strong fungicidal activity after 72 h treatment. These results indicated that **Ir2** combined with FLC killed *C. albicans* SC5314FR directly.

### 2.4 Ir2 combined with FLC inhibit the formation of *C. albicans* SC5314FR biofilm

**Figure 3:**
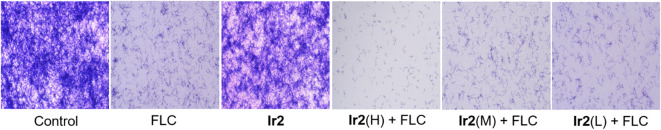
Effect of **Ir2** combined with FLC on biofilm formation of drug-resistant *C. albicans* SC5314FR (20 ×). *C. albicans* SC5314FR was incubated with FLC, **Ir2** or **Ir2** + FLC at 37 °C for 24 h, and the biofilm mass was determined by crystal violet staining.

The formation of biofilm is a major virulence factor of *C. albicans.* In fact, a tightly arranged fungal cell community is extremely difficult to eradicate, which is the main reason for the resistance of commonly used antifungal drugs (Ponde et al., 2021). For this reason, we chose **Ir2** combined with FLC to inhibit the formation of biofilm. The results showed that **Ir2** alone could not inhibit the biofilm formation of *C. albicans* SC5314FR, while FLC had a certain inhibitory effect on biofilm formation. However, when two drugs were used together, the biofilm formation was more obviously inhibited in a dose-dependent manner (**Figure 3**).

### 2.5 Ir2 combined with FLC inhibit the hyphal formation of *C. albicans* SC5314FR

**Figure 4:**
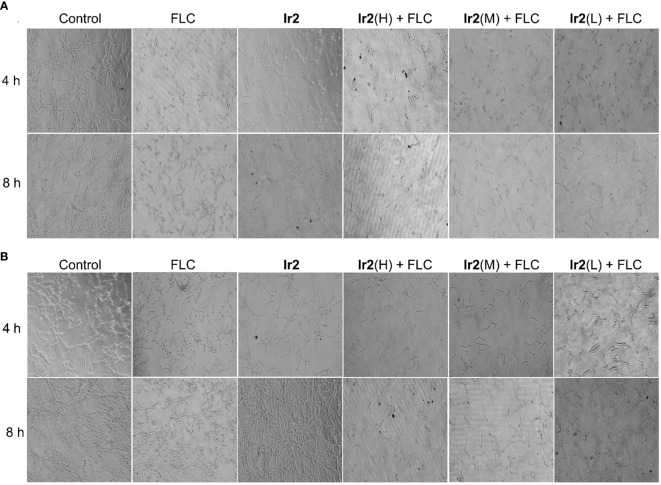
Effects of the combination of **Ir2** and FLC on hyphal morphological transformation. **(A)** Spider liquid medium, **(B)** SD + 10% FBS (20 ×). *C. albicans* SC5314FR was incubated with FLC, **Ir2** or **Ir2** + FLC at 37 °C for 4 h or 8 h, and the mycelia of different groups were observed under an inverted microscope for growth and photographed.

When *C. albicans* changed from yeast state to hyphal state, its virulence would be changed, which was the main cause of its disease. To this end, we evaluated the effect of the combination of **Ir2** and FLC on the morphological transformation of hyphae of drug-resistant *C. albicans* SC5314FR through hypha formation experiments. The results showed (**Figures 4A, B**) that the drug-resistant strain SC5314FR had formed complex, elongated, and coiled hyphae after 8 h of culture in two different hypha induction media, Synthetic Dropout medium (SD) (containing 10% fetal bovine serum (FBS)) and Spider medium. After FLC treatment, it inhibited the mycelia growth to a certain extent at 4 h, but it could not inhibit the formation of complex mycelia at 8 h. However, **Ir2** alone did not inhibit the formation of hypha. In contrast to FLC, the combination of **Ir2** and FLC inhibited hyphal formation in a dose-dependent manner. These results indicated that the combined use of the two drugs reduced the formation of hyphae of drug-resistant *C. albicans* and its virulence. We further detected the effect of drug combinations on the expression of virulence genes related to *C. albicans* SC5314FR hyphae using PCR experiments. The data (**Figure S13**) showed that their impact on these gene expressions was not significant. So, we next explored the effects of **Ir2** combined with FLC on intracellular reactive oxygen species (ROS), mitochondrial membrane potential (MMP) and ATP levels.

### 2.6 Ir2 combined with FLC promote the accumulation of intracellular ROS, and decrease MMP and ATP levels

ROS is often accompanied by host's phagocytosis against *C. albicans*. It can cause oxidative stress damage by interacting with the protein of the strain and induce the programmed cell death of the strain (Lv et al., 2022; Ma et al., 2023a). In the cell, ROS is mainly produced by mitochondria, and the rate of ROS production by mitochondria is mainly regulated by the transmembrane potential of mitochondrial inner membrane (Peng et al., 2022; Campos et al., 2023). Therefore, we detected the effect of **Ir2** combined with FLC on intracellular ROS and mitochondrial MMP of drug-resistant *C. albicans* by fluorescent probe 2',7'-dichlorofluorescein diacetate (DCFH-DA) and 5,5',6,6'-tetrachloro-1,1',3,3'-tetraethylbenzimidazolylcarbocyanine iodide (JC-1). The results showed that the combination of **Ir2** and FLC promoted the accumulation of intracellular ROS (**Figure 1A**) and decreased MMP (**Figure 1B**) in a dose-dependent manner. Therefore, we speculated that the combination of **Ir2** and FLC caused the mitochondrial damage of drug-resistant *C. albicans*. As the main production of ROS, the damaged mitochondria caused a large amount of ROS accumulation, and then caused the oxidative stress reaction of *C. albicans*, resulting in programmed death of drug-resistant *C. albicans*. In addition, the decrease in ATP levels is closely related to mitochondrial dysfunction. Therefore, we determined the ATP production of *C. albicans*. The results showed that **Ir2** or FLC alone could not reduce the ATP production by mitochondria of *C. albicans*, and the combination of **Ir2** and FLC significantly reduced the ATP production by *C. albicans* in a dose-dependent manner (**Figure 1C**).

## 3 Conclusion

In summary, we evaluated the antifungal activity of a series of tpphz modified cyclometalated iridium(III) and polypyridyl ruthenium(II) complexes against *C. albicans*. The serial compounds combined with FLC have antifungal activity on *C. albicans*, wherein **Ir2** and FLC in combination have significant antifungal activities on drug resistant and sensitive strains of *C. albicans in vitro*. The combination of the two drugs can inhibit the time growth curve to directly kill the *C. albicans*; it also inhibits the formation of biofilm and hypha. In addition, the combination of the two drugs reduced the MMP of drug-resistant *C. albicans*, causing mitochondrial damage and sharp increase in the accumulation of ROS. To sum up, the combination of **Ir2** and FLC can kill drug resistant *C. albicans* probably by damaging mitochondria. It is expected to become an effective strategy to solve the clinical resistance of *C. albicans* and optimize the treatment plan of clinical fungal infection.

## 4 Materials and methods

### 4.1 Materials and instruments

IrCl_3_·nH_2_O (J&K), ppy (J&K), thpy (J&K), dfppy (J&K), RuCl_3_·nH_2_O (J&K), bpy (J&K), phen (J&K), DIP (J&K), 1,10-phenanthroline-5,6-dione (J&K), 5,6-diamino-1,10-phenanthroline (J&K), FLC (Nanchang Hongyi Pharmaceutical Co., Ltd.), enzyme-labeled instrument (Nanjing Detie Test Equipment Co., Ltd.), Sabouraud's Dextrose Brother (HKM), Sabouraud Dextrose Agar (HKM), FBS (VivaCell), ROS Assay Kit (BRYOTIME), MMP Assay Kit with JC-1 (BRYOTIME). FLC and the compounds were dissolved in dimethyl sulfoxide (DMSO) before the experiment, and the concentration of DMSO in the experiment was less than 1%.

### 4.2 Preparation of Ir1-Ir3 and Ru1-Ru3

[Ir(ppy)_2_(tpphz)](PF_6_) (**Ir1**) (Chen et al., 2011), [Ir(dfppy)_2_(tpphz)](PF_6_) (**Ir3**) (Cho et al., 2016), [Ru(bpy)_2_(tpphz)](PF_6_)_2_ (**Ru1**) (Gill et al., 2011), [Ru(phen)_2_(tpphz)](PF_6_)_2_ (**Ru2**) (Gill et al., 2011) and [Ru(DIP)_2_(tpphz)](PF_6_)_2_ (**Ru3**) (Alatrash et al., 2017) were synthesized according the literatures.

[Ir(ppy)_2_(tpphz)](PF_6_) (**Ir1**): ^1^H NMR (600 MHz, DMSO-*d*_6_) δ 9.62 (dd, *J* = 37.0, 7.4 Hz, 4H), 8.65 (s, 2H), 8.37 (dd, *J* = 32.9, 6.3 Hz, 4H), 8.20 (dd, *J* = 7.9, 5.1 Hz, 2H), 8.04 (d, *J* = 7.9 Hz, 2H), 7.95 – 7.80 (m, 6H), 7.16 – 7.01 (m, 6H), 6.40 – 6.35 (m, 2H). ESI-HRMS (CH_3_OH): *m/z* 885.2041 [M-PF_6_]^+^.

[Ir(thpy)_2_(tpphz)](PF_6_) (**Ir2**): The synthetic route of **Ir2** was shown **Scheme 2**. The mixture of 1,10-phenanthroline-5,6-dione (2.1 equiv.) and the precursor of cyclometalated iridium(III) [Ir(thpy)_2_Cl]_2_ (1.0 equiv.) was firstly refluxed in CH_2_Cl_2_/CH_3_OH for 4 h. Then, 5,6-diamino-1,10-phenanthroline (2.0 equiv.) in 10 mL CH_3_OH were added and refluxed for 6 h under nitrogen. After cooling to room temperature, the solvent was evaporated and the crude product was chromatographed over silica gel by using CH_2_Cl_2_/CH_3_OH (10/1, v/v) as an eluent to obtain **Ir2**. Yield: 38% (yellow solid). ^1^H NMR (600 MHz, DMSO-*d*_6_) δ 9.60 (dd, *J* = 42.2, 7.6 Hz, 4H), 8.62 (s, 2H), 8.37 (d, *J* = 4.3 Hz, 2H), 8.25 – 8.21 (m, 2H), 7.81 (dt, *J* = 12.2, 6.3 Hz, 10H), 6.86 (t, *J* = 6.1 Hz, 2H), 6.35 (d, *J* = 4.7 Hz, 2H). ESI-HRMS (CH_3_OH): *m/z* 897.1188 [M-PF_6_]^+^. Elemental analysis: calcd (%) for C_42_H_24_F_6_IrN_8_PS_2_: C, 48.41; H, 2.32; N, 10.75; found: C, 48.58; H, 2.40; N, 10.90.

**Scheme 1:**
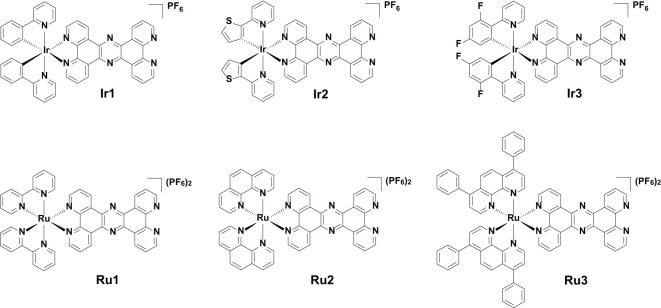
Chemical structures of [Ir(C-N)_2_(tpphz)](PF_6_) (**Ir1**-**Ir3**) and [Ru(N-N)_2_(tpphz)](PF_6_)_2_ (**Ru1**-**Ru3**).

**Scheme 2:**
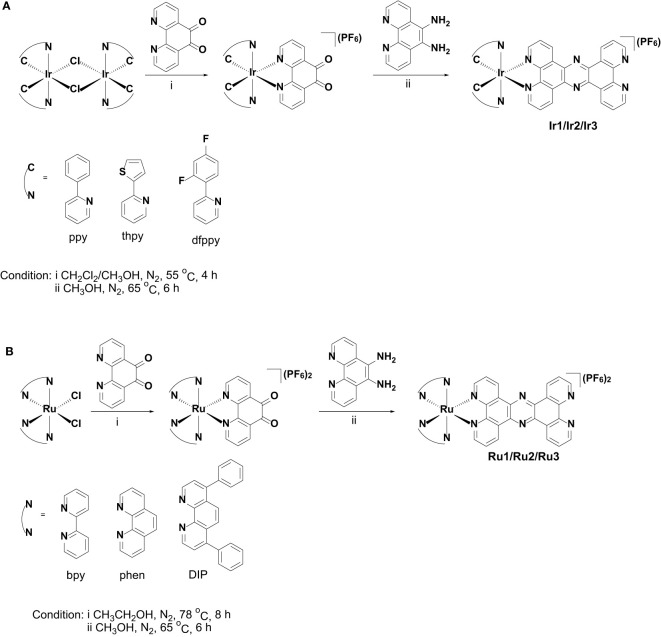
Synthetic routes of **Ir1**-**Ir3 (A)** and **Ru1**-**Ru3 (B)**.

[Ir(dfppy)_2_(tpphz)](PF_6_) (**Ir3**): ^1^H NMR (600 MHz, DMSO-*d*_6_) δ 9.68 (dd, *J* = 45.7, 7.6 Hz, 4H), 8.66 (s, 2H), 8.50 (dd, *J* = 5.0, 0.9 Hz, 2H), 8.37 (d, *J* = 8.9 Hz, 2H), 8.21 (dd, *J* = 8.1, 5.1 Hz, 2H), 8.07 – 7.95 (m, 4H), 7.87 (dd, *J* = 7.8, 4.2 Hz, 2H), 7.18 – 7.08 (m, 4H), 5.80 (dd, *J* = 8.2, 2.2 Hz, 2H). ESI-HRMS (CH_3_OH): *m/z* 957.1691 [M-PF_6_]^+^.

[Ru(bpy)_2_(tpphz)](PF_6_)_2_ (**Ru1**): ^1^H NMR (600 MHz, DMSO-*d*_6_) δ 9.61 (dd, *J* = 62.9, 7.0 Hz, 4H), 8.93 (dd, *J* = 21.8, 8.1 Hz, 4H), 8.63 (s, 2H), 8.36 – 8.26 (m, 4H), 8.18 – 8.01 (m, 6H), 7.91 (dd, *J* = 10.9, 4.6 Hz, 4H), 7.68 – 7.64 (m, 2H), 7.43 (t, *J* = 6.1 Hz, 2H). ESI-HRMS (CH_3_OH): *m/z* 399.0774 [M-2PF_6_]^2+^, 943.1164 [M-PF_6_]^+^.

[Ru(phen)_2_(tpphz)](PF_6_)_2_ (**Ru2**): ^1^H NMR (600 MHz, DMSO-*d*_6_) δ 9.61 (d, *J* = 7.0 Hz, 2H), 9.41 (d, *J* = 6.3 Hz, 2H), 8.87 – 8.68 (m, 6H), 8.45 (s, 6H), 8.28 (d, *J* = 4.6 Hz, 2H), 8.16 (d, *J* = 4.5 Hz, 2H), 7.86 (dt, *J* = 20.9, 6.1 Hz, 8H). ESI-HRMS (CH_3_OH): *m/z* 423.0709 [M-2PF_6_]^2+^, 991.1191 [M-PF_6_]^+^.

[Ru(DIP)_2_(tpphz)](PF_6_)_2_ (**Ru3**): ^1^H NMR (600 MHz, DMSO-*d*_6_) δ 9.85 – 9.45 (m, 4H), 8.75 (s, 1H), 8.62 (s, 1H), 8.44 (dd, *J* = 30.5, 5.0 Hz, 4H), 8.33 (s, 4H), 8.05 – 7.97 (m, 2H), 7.87 (dd, *J* = 34.2, 5.3 Hz, 6H), 7.66 (dddd, *J* = 53.2, 23.0, 14.2, 6.9 Hz, 22H). ESI-HRMS (CH_3_OH): *m/z* 575.1413 [M-2PF_6_]^2+^, 1295.2459 [M-PF_6_]^+^.

### 4.3 *C. albicans* strain and culture condition

The sensitive strain, *C. albicans* SC5314, was purchased from the American type culture collection. SC5314, ATCC14053 and ATCC10231 FLC-resistant strain (SC5314FR, ATCC14053FR and ATCC10231FR): sensitive strain SC5314, ATCC14053 and ATCC10231 were induced by FLC to be resistant to FLC. *C. albicans* CA23 and CA556, two clinical isolates, were presented by Professor Li Yuye, Department of Dermatology and Sexology, the First Affiliated Hospital of Kunming Medical University (Yunnan, China).

### 4.4 Determination of MIC: microdilution method

The MIC of a combination of **Ir1**-**Ir3** and **Ru1**-**Ru3** with FLC against *C. albicans* was determined using microdilution method. In short, the incubated *C. albicans* strains were scraped into Sabouraud's Dextrose Broth (SDB) and prepared into fungal suspension with the final concentration of 1 × 10^5^ colony-forming units (CFU)/mL. The corresponding drugs were added according to the groups. After 24 h, the absorbance (OD 630 nm) of each group was measured with a microplate reader, and the MIC and FICI of each group were calculated. When FICI was ≤ 0.5, the two drugs interacted synergistically. When 0.5 < FICI ≤ 4, the mode of action of the two drugs was irrelevant. When FICI > 4, the action mode of the two drugs was antagonism.

### 4.5 Research on time-kill curve

A proper amount of *C. albicans* was scraped into fresh SDB, mixed and counted until the final concentration was 1 × 10^5^ CFU/mL. The corresponding drugs were added according to the groups, and cultured in a constant temperature shaker at 37 °C and with shaking at 150 rpm. 100 μL samples were collected in the ultra-clean benches at time points 0, 4, 8, 12, 24, 36, 48 and 72 h. Three wells were plated in each group. The OD value at 625 nm wavelength was detected by enzyme-labeled instrument (where FLC concentration was 3 μg/mL, **Ir2** used alone was 3 μg/mL, and the combination was **Ir2**(H) + FLC: 6 μg/mL + 3μg/mL, **Ir2**(M) + FLC: 3 μg/mL + 3 μg/mL, **Ir2**(L) + FLC: 1.5 μg/mL + 3 μg/mL). The concentration of drugs used in subsequent experiments was subject to this standard.

### 4.6 *C. albicans* biofilm formation experiment

The drug-resistant *C. albicans* SC5314FR was dissolved in RPMI-1640 + 10% FBS medium, and the fungal concentration was adjusted to 1 × 10^5^ CFU/mL. They were transferred to 24-well plates with 1 mL per well, and cultured in a constant temperature and humidity box at 37 °C for 90 min. The supernatant was sucked and discarded. According to the grouping, 1 mL of fresh 1640 + 10% FBS medium with or without drug was added, and cultured in a constant temperature and humidity box at 37 °C for 24 h, and the supernatant was sucked and discarded. Then 400 μL 0.5% crystal violet was added for staining for 30 min, followed by PBS washing for three times, and then 400 μL PBS was added for observation and photographing under an inverted microscope.

### 4.7 Study on morphological transformation of *C. albicans* mycelium

*C. albicans* SC5314FR was dissolved in SD + 10% FBS and Spider liquid medium, and the final concentration was adjusted to 1 × 10^5^ CFU/mL. The corresponding drugs were added in groups, and 1 mL of fungal suspension with or without drug was added into each well of 24-well plates. The samples were cultured in a constant temperature and humidity box at 37 °C. After 4 and 8 h, the mycelia of different groups were observed under an inverted microscope for growth and photographed.

### 4.8 Detection of intracellular ROS of *C. albicans*

A proper amount of drug-resistant *C. albicans* SC5314FR was scraped into SDB. And the *C. albicans* cell suspension was prepared according to the groups, adjusted to the medium concentration of 1 × 10^5^ CFU/mL. Under the dark condition, fluorescent probe DCFH-DA was added and incubated for 30 min in the dark. After being washed and re-suspended with PBS again, they were mixed evenly and absorbed 100 μL into a black 96-well plates, with three wells in each group. The fluorescence value was measured by the full-wavelength multifunctional enzyme-labeled instrument (excitation wavelength: 488 nm, emission wavelength: 525 nm).

### 4.9 Detection of MMP of *C. albicans*

A proper amount of drug-resistant *C. albicans* SC5314FR was scraped into SDB, mixed and counted, the concentration of fungal suspension was 1 × 10^5^ CFU/mL. The corresponding drugs were added according to groups, cultured with a constant temperature shaking table for 16 h. Then JC-1 working solution was added. After uniform mixing, the samples were incubated in a constant-temperature shaking table for 20 min, and the supernatant was discarded after centrifugation. After being washed and re-suspended with JC-1 staining buffer, 100 μL fungal suspension was collected into a black 96-well plate with three re-wells in each group. The red (excitation wavelength: 488 nm, emission wavelength: 590 nm) and green fluorescence values (excitation wavelength: 488 nm, emission wavelength: 530 nm) were measured by the full-wavelength multifunctional enzyme-labeled instrument. The MMP was determined by the ratio of the red fluorescence value to the green fluorescence value.

### 4.10 Detection of ATP of *C. albicans*

The intracellular ATP production was measured using ATP assay kits (Beyotime Institute of Biotechnology, Haimen, China) according to the manufacturer’s instructions. Briefly, the concentration of *C. albicans* SC5314FR in medium were adjusted to 1 × 10^5^ CFU/mL, and the corresponding drugs were added according to groups, cultured at 37°C for 16 h. Cells were then collected and washed with ice-cold PBS. ATP levels in cells were calculated according to the standard curve. The results were expressed in nmol/mg protein.

### 4.11 Statistical analysis

Biological experiments were repeated at least 3 times and the results were presented as mean ± standard deviation.

## Data availability statement

The original contributions presented in the study are included in the article/**Supplementary Material**. Further inquiries can be directed to the corresponding authors.

## Author contributions

J-JL, X-RM, HZ, and L-YZ contributed to the synthesis and characterization of complexes. Z-CX contributed to the antifungal activity and mechanism, as well as the writing of article. R-RW, R-TL, and R-RY designed the project. All authors contributed to the article and approved the submitted version.
