# Supplementary material for: Cyclometalated iridium(III) complexes combined with fluconazole: antifungal activity against resistant C. albicans
Source: Front Cell Infect Microbiol. 2023 Jul 21;13:1200747. doi: 10.3389/fcimb.2023.1200747 (PMC10401479; doi:10.3389/fcimb.2023.1200747)
Supplement: Supplementary file 1 [file DataSheet_1.docx]

Supplementary Material

Cyclometalated iridium(III) complexes combined with fluconazole: antifungal activity against resistant *C. albicans*

Jun-Jian Lu ^a,1^, Zhi-Chang Xu ^b,1^, Hou Zhu ^a^, Lin-Yuan Zhu ^a^, Xiu-Rong Ma ^a^, Rui-Rui Wang^b*^, Rong-Tao Li ^a^, Rui-Rong Ye^a*^

*^a^* Faculty of Life Science and Technology, Kunming University of Science and Technology, Kunming 650500, P. R. China

*^b^* College of Chinese Materia Medica, Yunnan University of Chinese Medicine, Kunming, 650500, China

*** Correspondence:**

Prof. Rui-Rui Wang

wangrryucm@126.com

Dr. Rui-Rong Ye
[yerr@mail2.sysu.edu.cn](mailto:yerr@mail2.sysu.edu.cn)

^1^ These authors contributed equally.

## Supplementary Figures

**
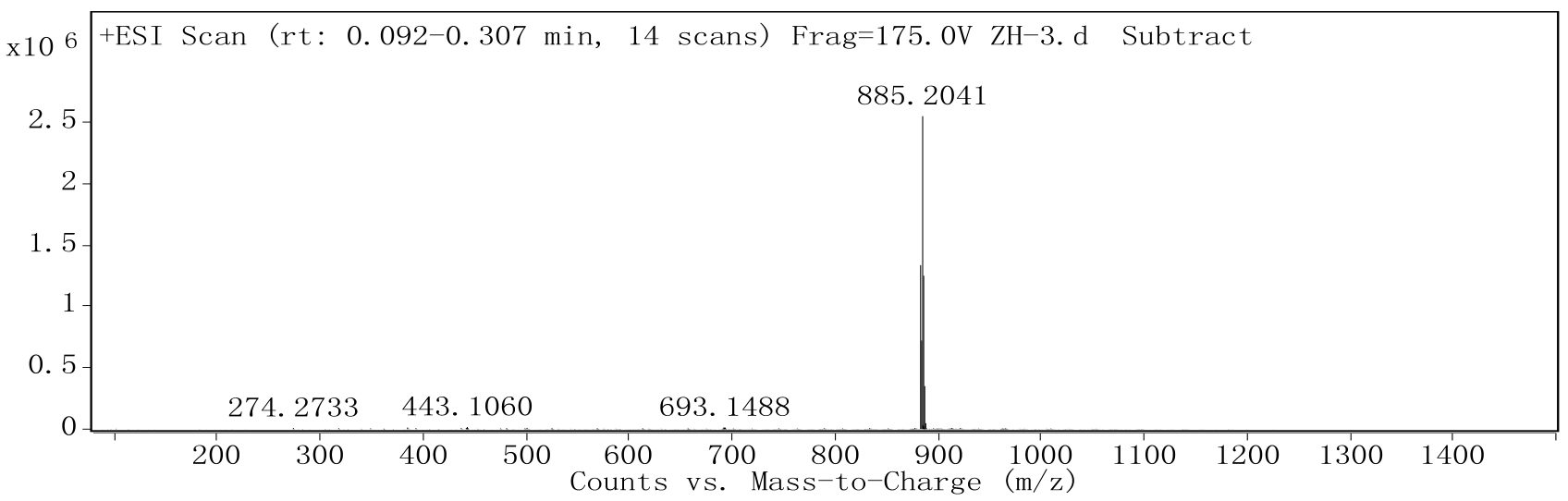
**

**Supplementary Figure 1.** ESI-HRMS characterization of **Ir1**, 885.2041 [M-PF_6_]^+^.


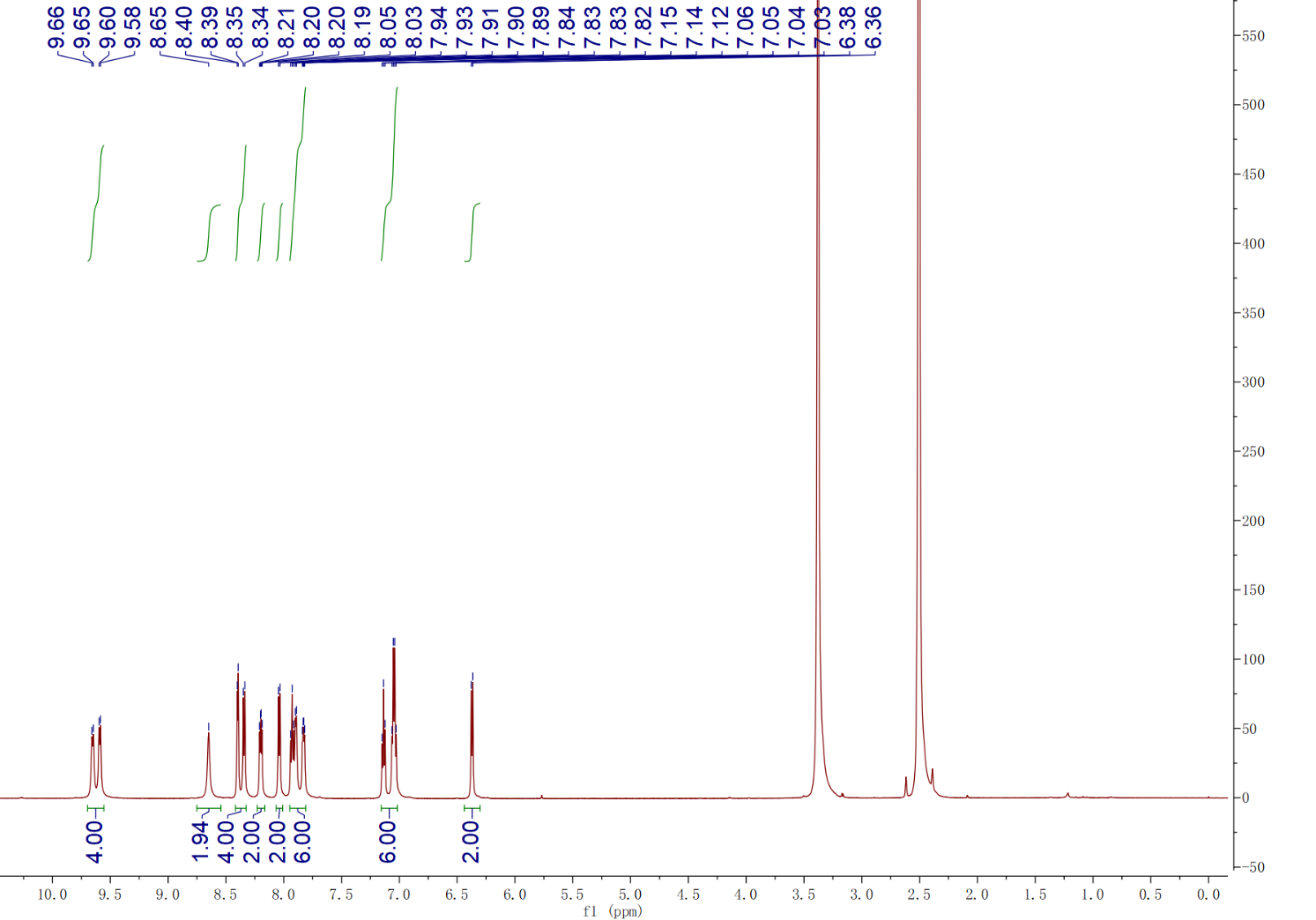


**Supplementary Figure 2.** ^1^H NMR spectrum of **Ir1**.


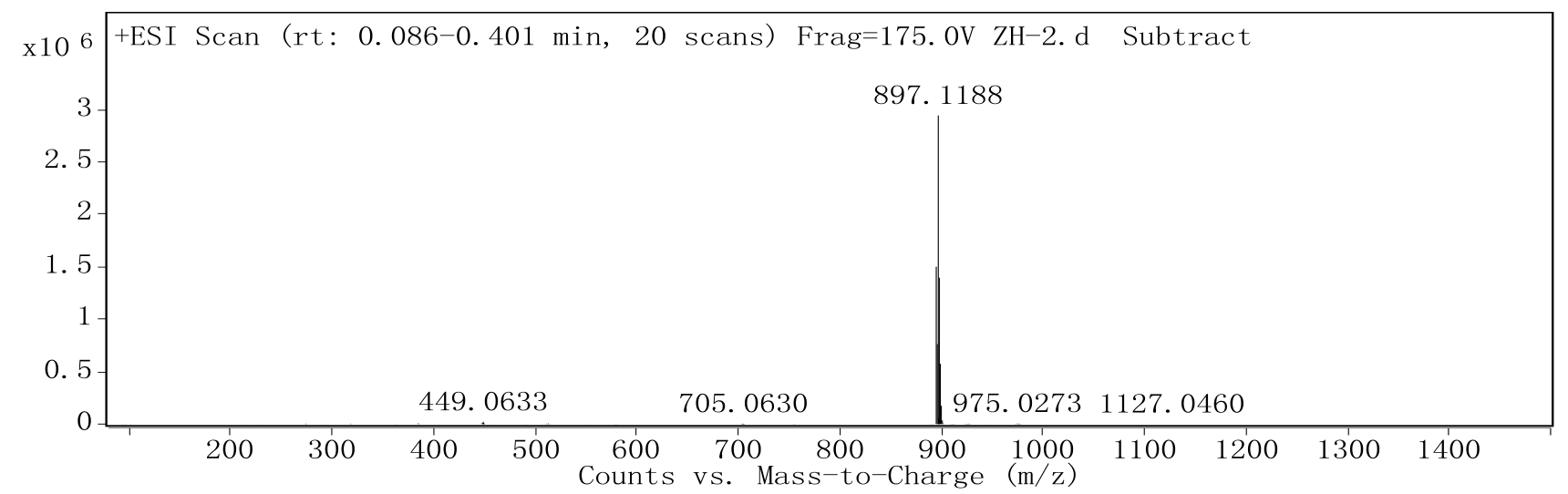


**Supplementary Figure 3.** ESI-HRMS characterization of **Ir2**, 897.1188 [M-PF_6_]^+^.


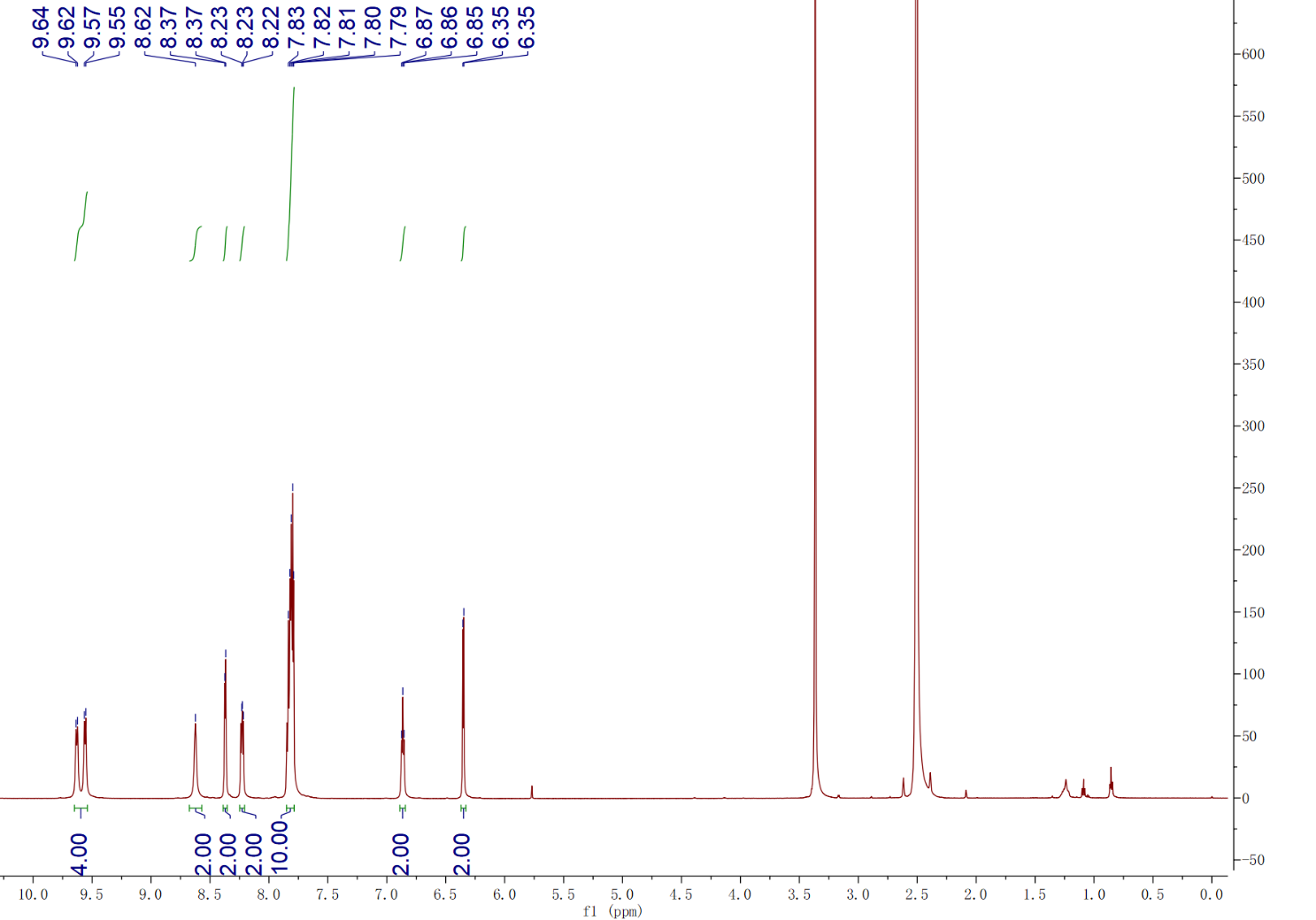


**Supplementary Figure 4.** ^1^H NMR spectrum of **Ir2**.

**
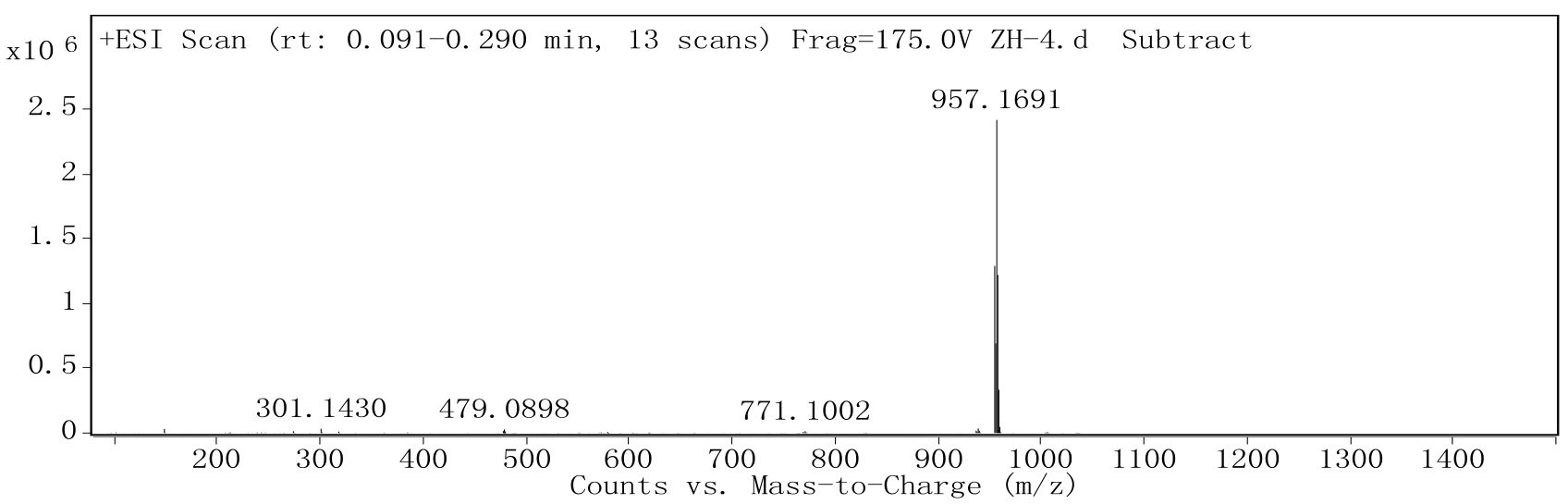
**

**Supplementary Figure 5.** ESI-HRMS characterization of **Ir3**, 957.1691 [M-PF_6_]^+^.


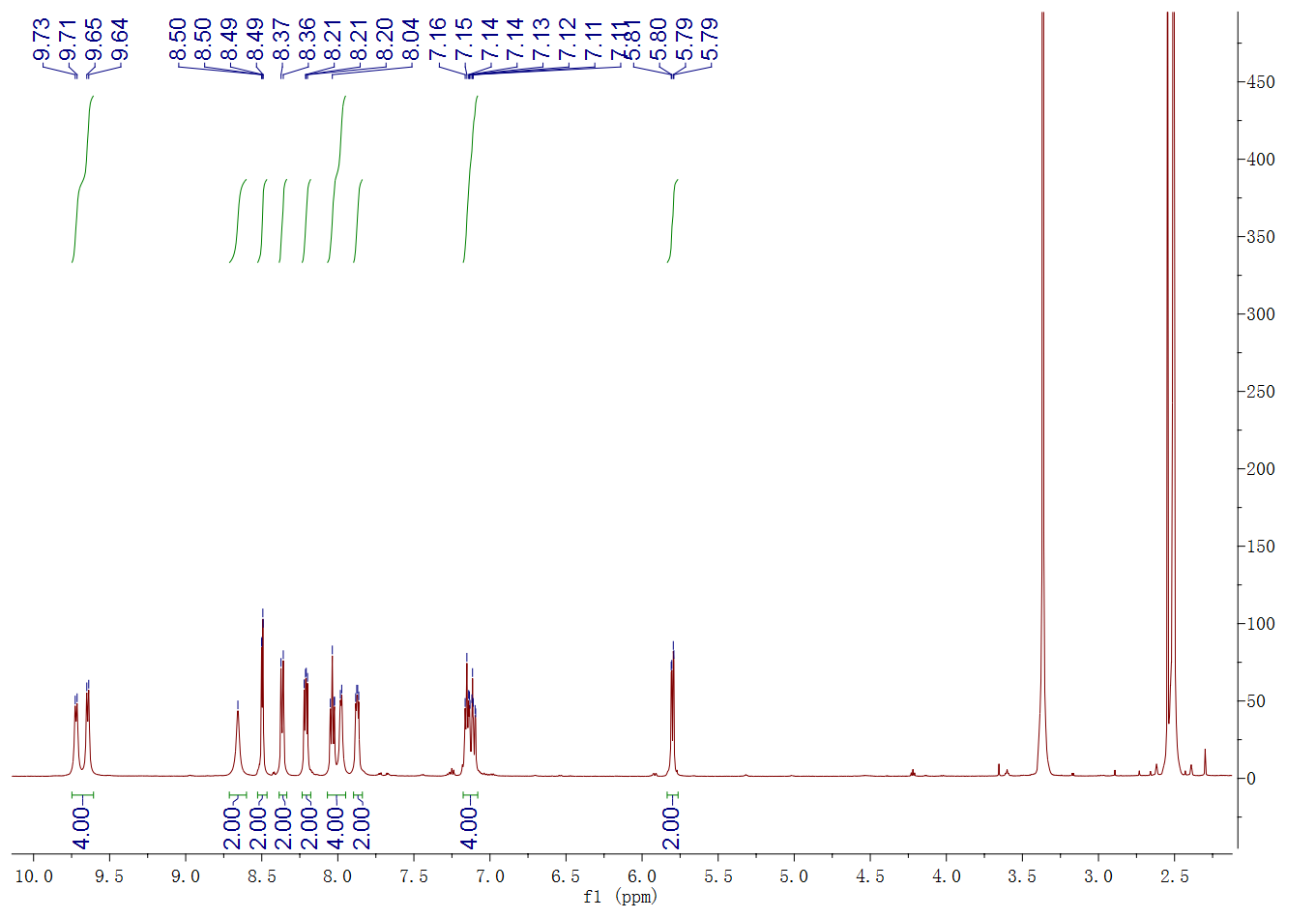


**Supplementary Figure 6.** ^1^H NMR spectrum of **Ir3**.

**
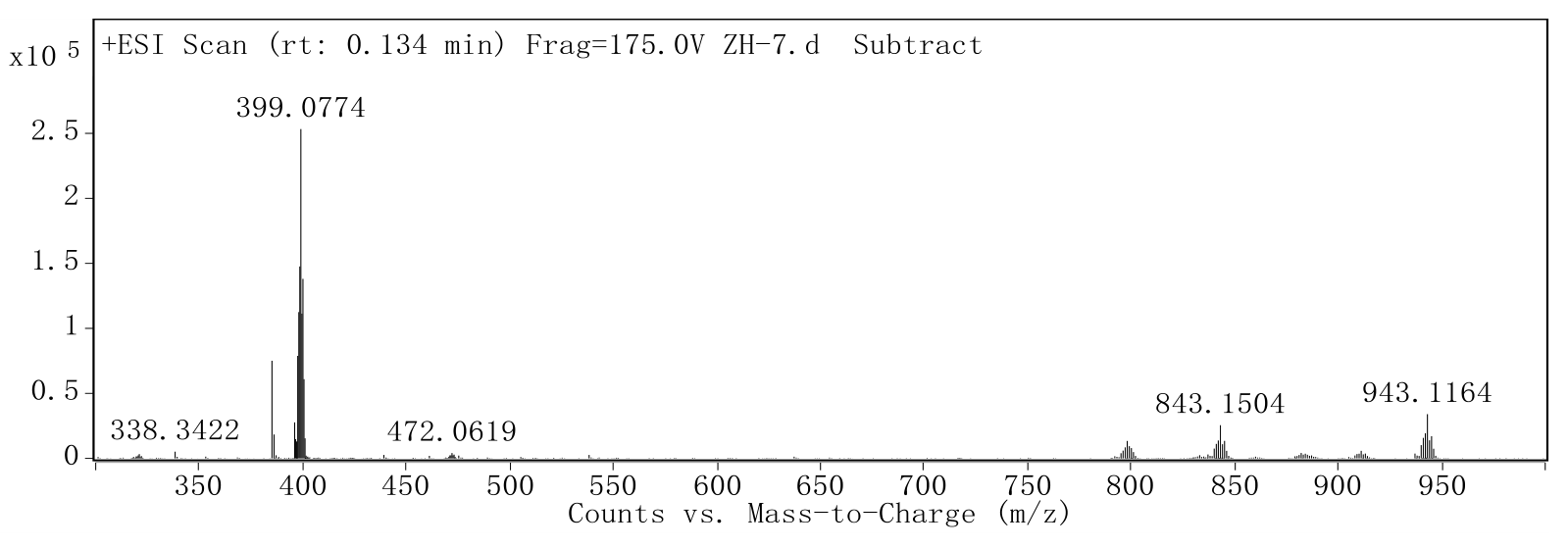
**

**Supplementary Figure 7.** ESI-HRMS characterization of **Ru1**, 399.0774 [M-2PF_6_]^2+^, 943.1164 [M-PF_6_]^+^.


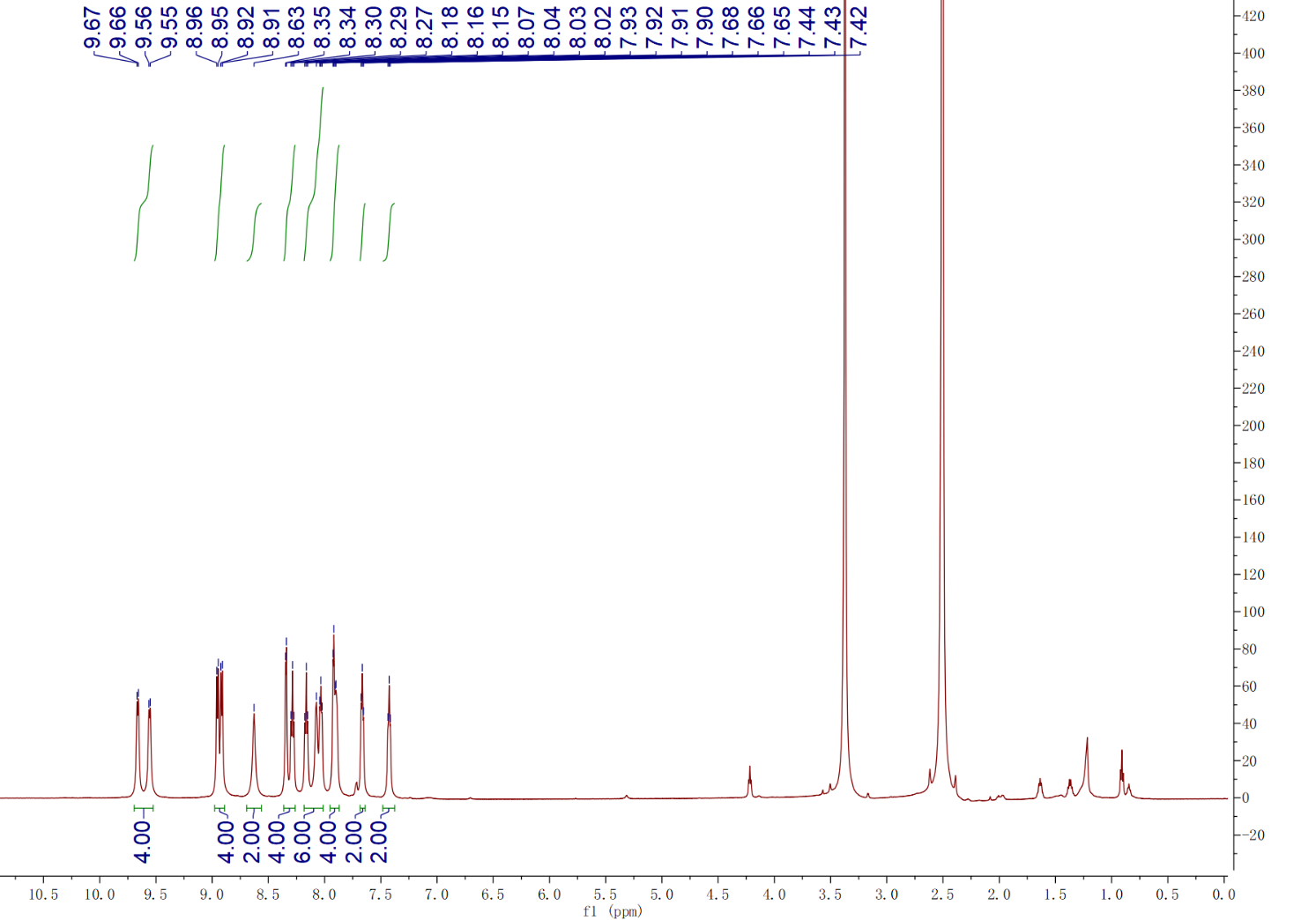


**Supplementary Figure 8.** ^1^H NMR spectrum of **Ru1**.

**
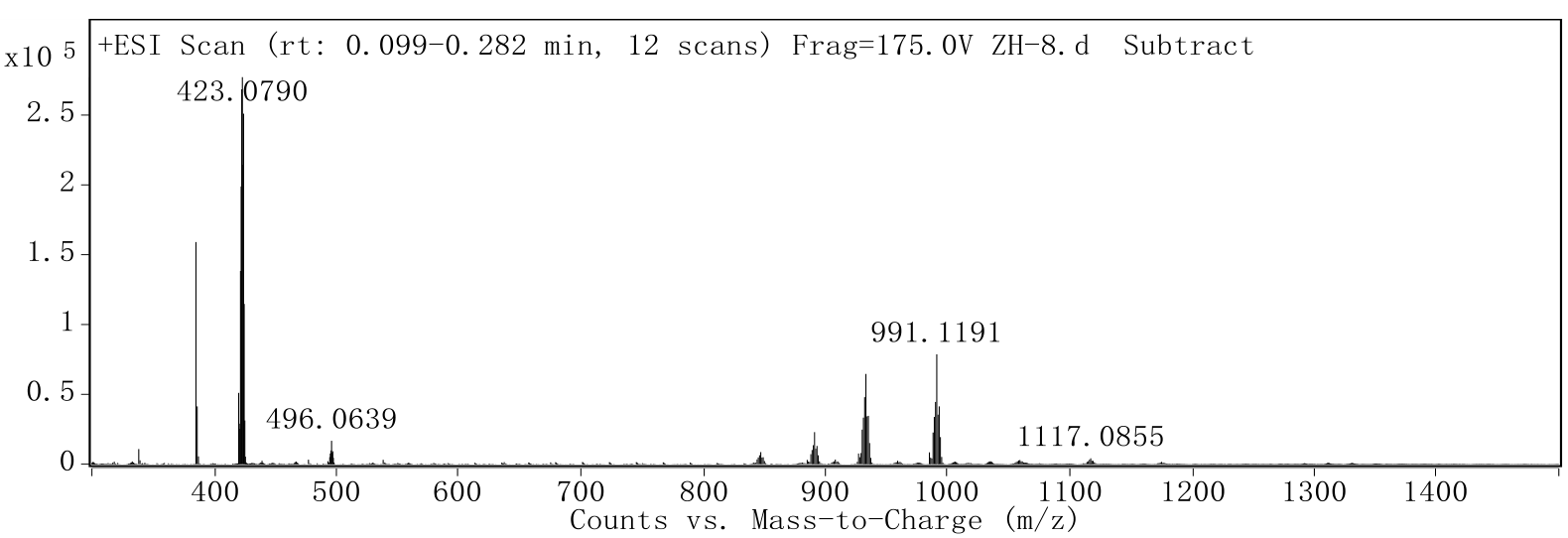
**

**Supplementary Figure 9.** ESI-HRMS characterization of **Ru2**, 423.0709 [M-2PF_6_]^2+^, 991.1191 [M-PF_6_]^+^.


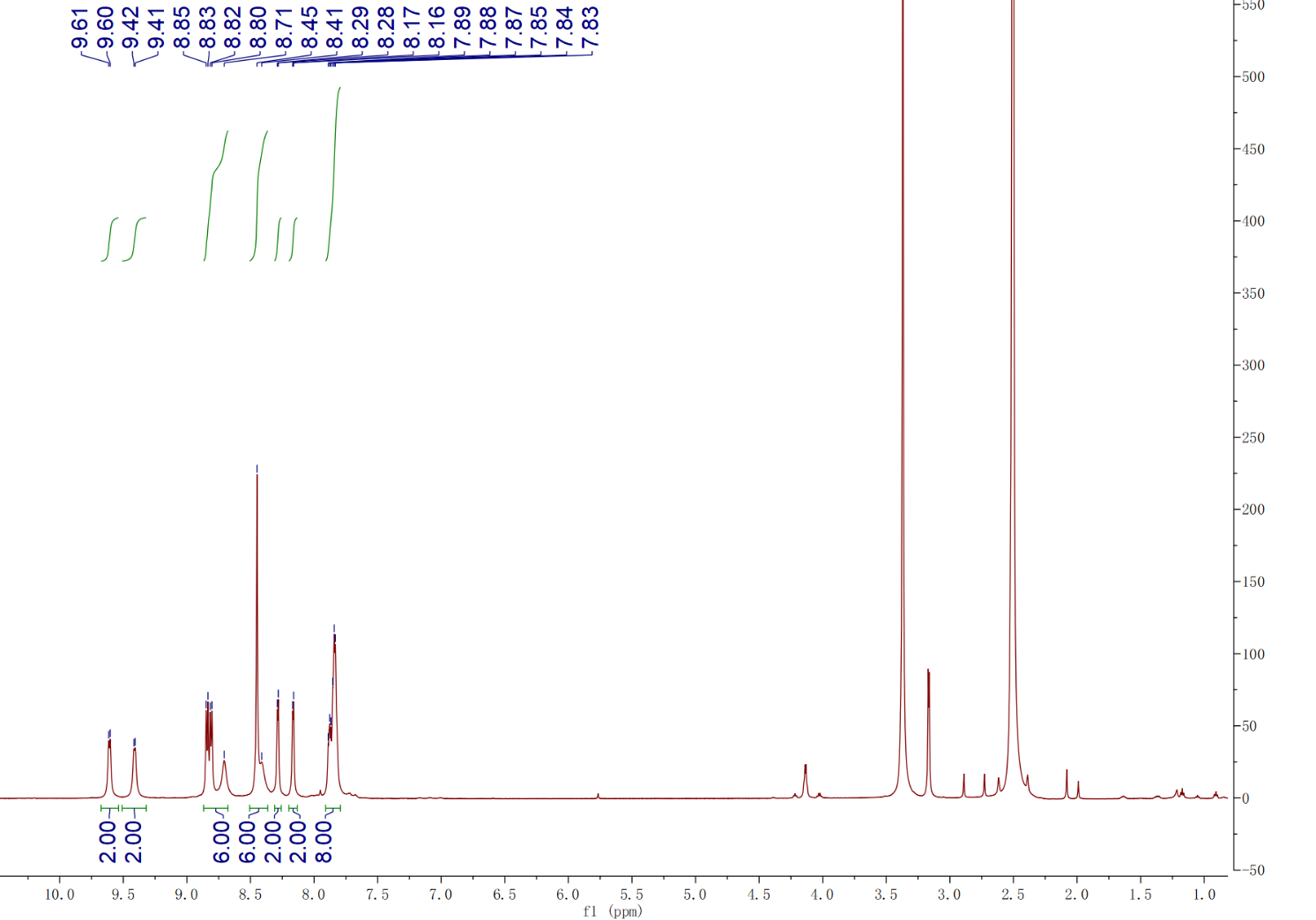


**Supplementary Figure 10.** ^1^H NMR spectrum of **Ru2**.

**
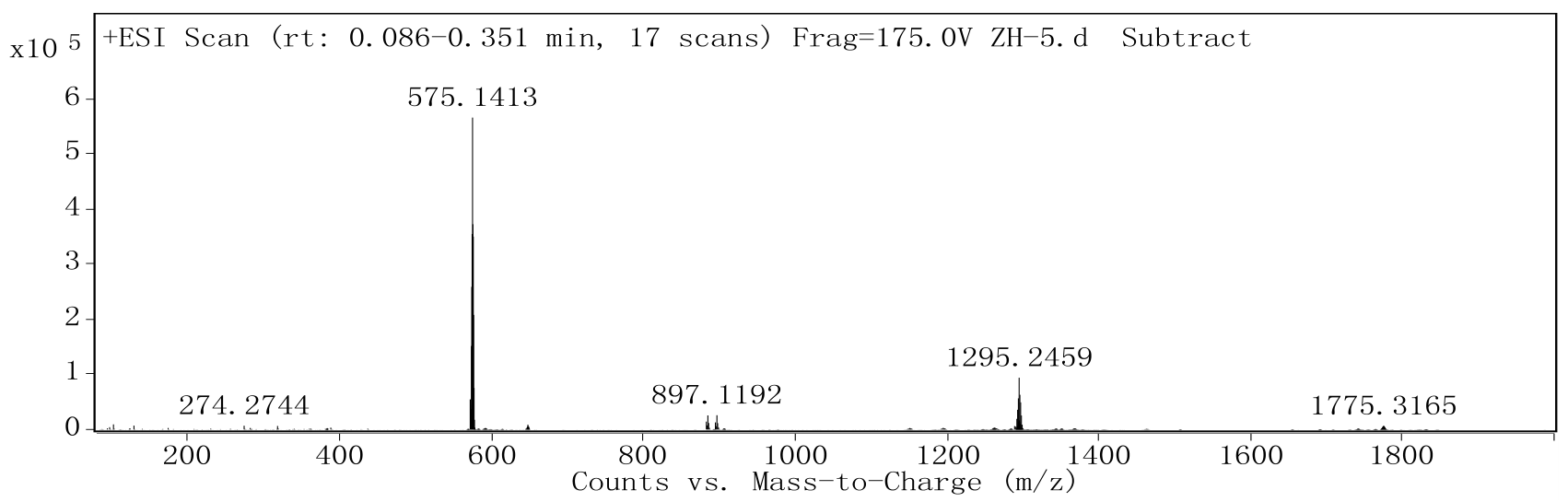
**

**Supplementary Figure 11.** ESI-HRMS characterization of **Ru3**, 575.1413 [M-2PF_6_]^2+^, 1295.2459 [M-PF_6_]^+^.


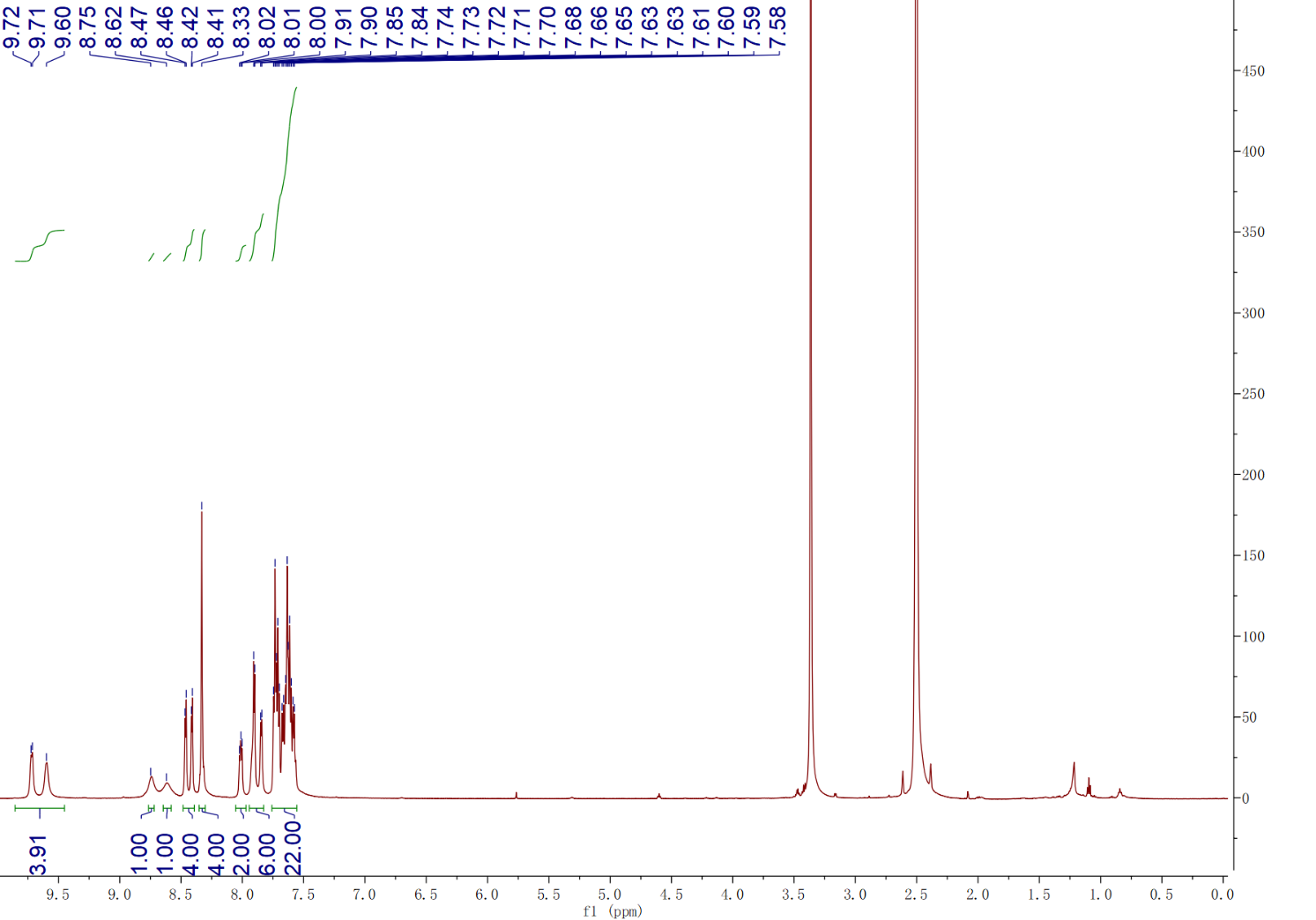


**Supplementary Figure 12.** ^1^H NMR spectrum of **Ru3**.


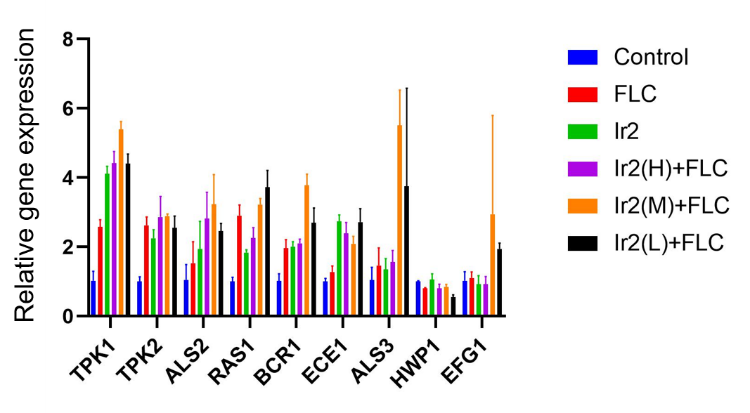


**Supplementary Figure 13.** The mRNA transcription levels of hyphae-related genes.
